# Study of Current Measurement Method Based on Circular Magnetic Field Sensing Array

**DOI:** 10.3390/s18051439

**Published:** 2018-05-05

**Authors:** Zhenhua Li, Siqiu Zhang, Zhengtian Wu, Ahmed Abu-Siada, Yuan Tao

**Affiliations:** 1 College of Electrical Engineering & New Energy, China Three Gorges University, Yichang 443002, China; zhangsiqiu1208@163.com (S.Z.); taoyuan2016ty@163.com (Y.T.); 2 Hubei Provincial Collaborative Innovation Center for New Energy Microgrid, China Three Gorges University, Yichang 443002, China; 3 School of Electronic and Information Engineering, Suzhou University of Science and Technology, Suzhou 215009, China; wzht8@mail.usts.edu.cn; 4 Electrical and Computer Engineering Department, Curtin University, Perth 6000, WA, Australia; A.AbuSiada@exchange.curtin.edu.au

**Keywords:** circular array, magnetic field sensor, current measurement, magnetic interference

## Abstract

Classic core-based instrument transformers are more prone to magnetic saturation. This affects the measurement accuracy of such transformers and limits their applications in measuring large direct current (DC). Moreover, protection and control systems may exhibit malfunctions due to such measurement errors. This paper presents a more accurate method for current measurement based on a circular magnetic field sensing array. The proposed measurement approach utilizes multiple hall sensors that are evenly distributed on a circle. The average value of all hall sensors is regarded as the final measurement. The calculation model is established in the case of magnetic field interference of the parallel wire, and the simulation results show that the error decreases significantly when the number of hall sensors *n* is greater than 8. The measurement error is less than 0.06% when the wire spacing is greater than 2.5 times the radius of the sensor array. A simulation study on the off-center primary conductor is conducted, and a kind of hall sensor compensation method is adopted to improve the accuracy. The simulation and test results indicate that the measurement error of the system is less than 0.1%.

## 1. Introduction

Instrument transformer is one of the key assets in the substations as it affirms the reliability of the protection and control systems [1,2,3,4]. The conventional magnetic core-based transformer is subject to saturation that limits its ability to measure direct current (DC) [5,6]. On the other hand, an electronic instrument transformer based on Rogowski coil is widely used in power systems due to its wide dynamic range and low cost. However, it cannot be used for DC measurement because of its sensing principle [7,8,9,10]. While an optical transformer can measure both alternating current (AC) and DC signals, the temperature stability and mechanical properties of optical crystals result in the low measurement accuracy of this instrument [11,12,13,14,15,16]. Commonly used methods for measuring DC current include shunt methods, magneto-resistance, and magneto-optical effects. However, the shunt methods are bulky, which renders them unsuitable for onsite applications. Furthermore, the accuracy of measurement methods based on giant magneto-resistance and magneto-optical effects is not high as it depends on the stability of optical and magnetic materials [17,18,19,20]. Owing to the rapid development of semiconductor materials and power electronic technology, magnetic field sensors, especially hall sensors, have attracted much attention for DC current measurement [21,22,23,24,25,26,27]. As there is no iron core, the hall sensor is not susceptible to magnetic saturation and can be used to measure large DC currents. 

To overcome the deficiency of conventional transformers and improve the accuracy of DC measurement methods, this paper proposes a current measurement method based on a circular magnetic field sensing array. Circular magnetic field sensing arrays were previously investigated in References [28,29]. However, these investigations mainly focused on signal processing algorithms. When there is the interference of an external current, an algorithm based on spatial Discrete Fourier Transform (DFT) was proposed to improve the interference rejection in Reference [28], and an algorithm based on the digital processing of a small set of magnetic field measurements was proposed to reduce the interference in Reference [29]. The method proposed in this paper is mainly focused on the sensor array structure and external interference factor, which improve the interference rejection by changing the number of sensors and the radius of sensor array. Moreover, a compensation method is proposed to improve the measurement accuracy when the conductor is off-center. The method employs eight hall sensors that are evenly distributed on a circle, in which the magnetic field strength can be measured and hence the current can be calculated. The interference from a parallel wire carrying the same current and an off-center wire are the dominant sources of error in practice. So, by using the average value of these hall sensors as the final measurement output, we can effectively reduce the external magnetic field interference and improve the accuracy. The simulation model is established and the simulation analysis is carried out when there is magnetic field interference from the parallel wire and off-center wire. The results show that the measurement error is less than 0.06% when the wire spacing is greater than 2.5 times the radius of the sensor array. By using a kind of compensation method for the hall sensor, the error generated by the off-center wire can be reduced effectively, and the whole system error is less than 0.1%.

## 2. Measurement Accuracy Analysis of Circular Magnetic Field Sensing Array

**Figure 1:**
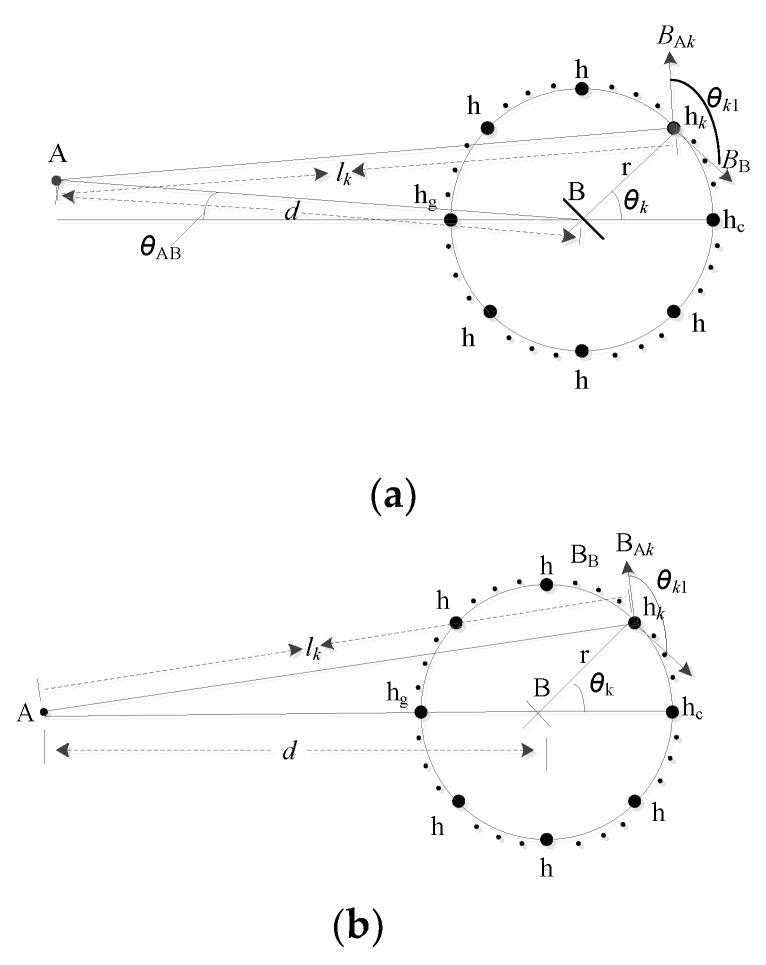
The magnetic field influence of a parallel conductor on the measurement accuracy of a circular hall sensors array: (**a**) conductor A, conductor B, h_g_, and h_c_ all are not on the same line; (**b**) conductor A, conductor B, h_g_, and h_c_ are on the same line.

The measurement method proposes several hall sensors distributed evenly on a circle, as shown in Figure 1. The average value of all employed hall sensors is considered as the final measurement output. This arrangement can effectively reduce the external magnetic field interference and improve the measurement accuracy. The detailed analysis of the magnetic field interference of a parallel conductor is presented below.

As shown in Figure 1, the distance between the conductors A and B is assumed to be *d*, while the currents in both conductors are assumed to be in opposite direction and of values *I*_A_ and *I*_B_, respectively. Conductor B, in which the current is to be measured, coincides with the center of the circular hall sensor array. *n*-hall sensors are evenly distributed on a circle of radius *r*. 

The hall sensors can only measure the magnetic field perpendicular to its sensitive area. The magnetic field density *B*_A*k*_ generated due to a current *I*_A_ passing in conductor A can be measured by the *k*th hall sensor and is given by:

(1) $$B_{Ak}=\frac{\mu_{0}I_{A}}{2\pi l_{k}}\cdot \cos\theta_{k1}$$

where:

(2) $$\left\{ \begin{array}{l} l_{k}=\sqrt{{\left(d\cos\theta_{AB}+r\cos\theta_{k}\right)}^{2}+{\left(r\sin\theta_{k}-d\sin\theta_{AB}\right)}^{2}}\\ \cos\theta_{k1}=-\frac{l_{k}{}^{2}+r^{2}-d^{2}}{2l_{k}r} \end{array}\right.$$

From which $\cos\theta_{k1}$ can be rewritten as:

(3) $$\cos\theta_{k1}=-\frac{r+d\cos(\theta_{k}+\theta )}{l_{k}}$$

$\left(\theta_{k}+\theta )\in (\theta ,\theta +2\pi \right)$, which means $\cos(\theta_{k}+\theta )$ and $r+d\cos(\theta_{k}+\theta )$ can be positive or negative; hence, $\cos\theta_{k1}$ can be positive or negative. Adopting the average method would reduce the influence of the current in conductor A on the overall readings of the *n* hall sensors.

The average magnetic field density value of *n* hall sensors due to a current in conductor A can be obtained from (1) and (3), as given below:

(4) $$B_{Aav}=-\frac{\mu_{0}I_{A}}{2\pi n}\sum_{k=1}^{n}\frac{d\cos(\theta_{k}+\theta )+r}{d^{2}+r^{2}+2dr\cos(\theta_{k}+\theta )}$$

The average magnetic field density due to a current *I*_B_ in conductor B can be given as:

(5) $$B_{Bav}=\frac{\mu_{0}I_{B}}{2\pi r}$$

The measurement error is:

(6) $$I_{e}=\frac{B_{Aav}}{B_{Bav}}=-\frac{rI_{A}}{nI_{B}}\sum_{k=1}^{n}\frac{d\cos(\theta_{k}+\theta )+r}{d^{2}+r^{2}+2dr\cos(\theta_{k}+\theta )}$$

The method proposed in Reference [28] is based on a spatial harmonic analysis of the magnetic field; the main content is about the analysis of the interference rejection algorithm. This method is useful to reduce the crosstalk relative error. It first calculates the magnetic scalar potential in a polar coordinate system, then the measurement error can be obtained by spatial Discrete Fourier Transform (DFT). In this paper, the main content concerns the error analysis of adjacent current, off-center influence, and the compensation method. From Equation (6), we determine that the measurement error is the ratio of the magnetic field generated due to the external current and the magnetic field generated due to the measured current. Moreover, the average method is adopted to reduce the interference of the external current.

When *I*_A_ and *I*_B_ are equal in magnitude:

(7) $$I_{e}=\frac{B_{Aav}}{B_{Bav}}=-\frac{r}{n}\sum_{k=1}^{n}\frac{d\cos(\theta_{k}+\theta )+r}{d^{2}+r^{2}+2dr\cos(\theta_{k}+\theta )}$$

In order to simplify the analysis, let the value of *θ* be 0°. Then:

(8) $$I_{e}=\frac{B_{Aav}}{B_{Bav}}=-\frac{r}{n}\sum_{k=1}^{n}\frac{d\cos\theta_{k}+r}{d^{2}+r^{2}+2dr\cos\theta_{k}}$$

where $\theta_{k}=\frac{2k\pi }{n},k=1,2\ldots ,n$.

The following simulation analysis is based on Equation (8).

### 2.1. Correlation of the Measurement Error and the Number of Hall Sensors

**Figure 2:**
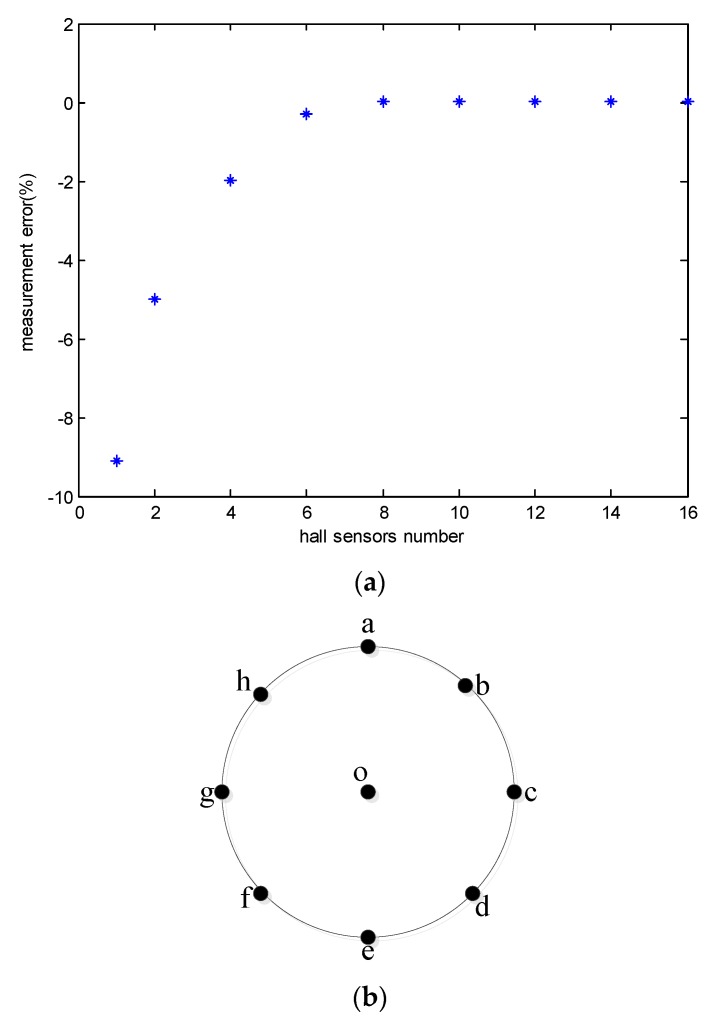
The measurement error and hall sensors position schematic: (**a**) the measurement error verses the hall sensors number; (**b**) the hall sensors position schematic. Hall sensors are distributed evenly in c and g with *n* = 2, in a, c, g, e with *n* = 4, and in a, b, c, d, e, f, g with *n* = 8.

Considering the physical configuration of two practical conductors, *r* is assumed to be in the range of 0.1 m–0.4 m and *d* in the range of 1 m–3 m. Simulation results for *r* = 0.1 m and *d* = 1.5 m are shown in Figure 2. It can be observed from Figure 2a that measurement error decreases substantially when the number of hall sensors increases. The error is less than 10^−8^ when *n* ≥ 8.

### 2.2. Correlation of the Measurement Error and the Distance between the Conductors

**Figure 3:**
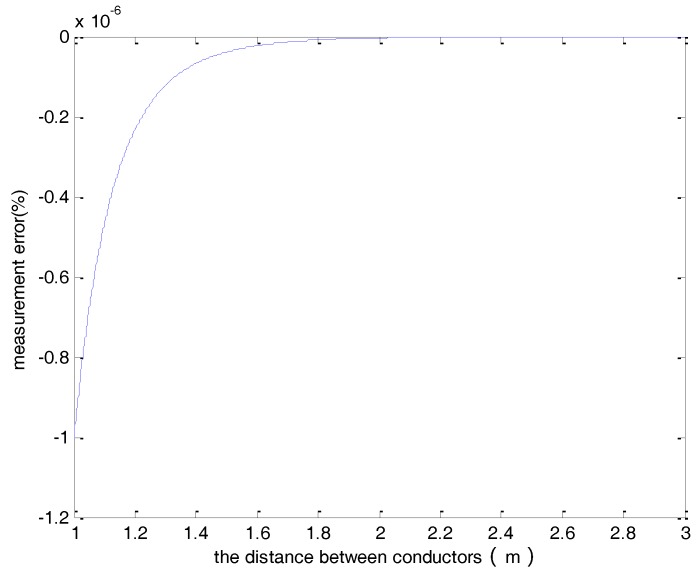
The measurement error verses the distance between the two conductors.

In this study, *r* is set to 0.1 m and *n* is chosen to be 8 based on the investigation of the previous section. Figure 3 shows the measurement error for a wide range of the distance between the two conductors (*d*). As can be seen in the figure, the error is less than 10^−8^ when the distance between the conductors is larger than 1 m.

### 2.3. Correlation of the Measurement Error and the Circle Radius

**Figure 4:**
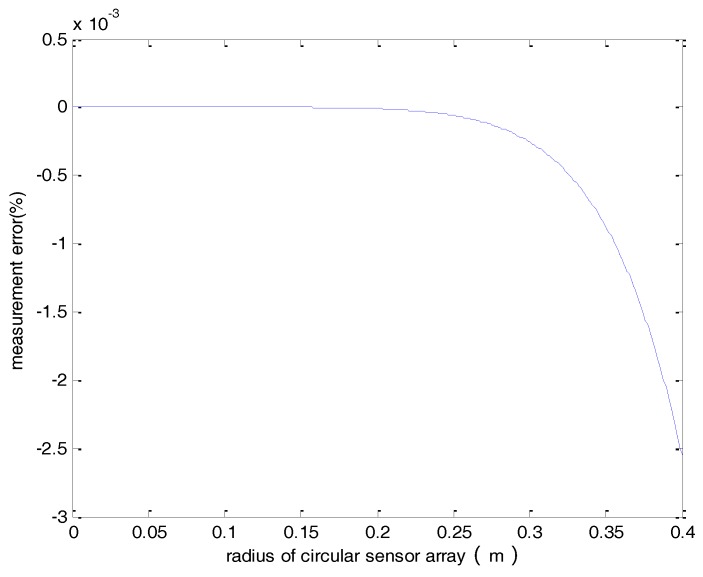
The measurement error verses the radius of the sensor array.

This analysis is conducted for *d* = 1.5 m and *n* = 8. Results shown in Figure 4 reveal that the measurement error is less than 0.003% for a circle radius less than 0.4 m and can be neglected.

### 2.4. Analysis of the Measurement Error with d and r

In order to facilitate the investigation of the influence of *r* and *d* on the measurement accuracy, Equation (8) is rewritten as:

(9) $$I_{e}=\frac{B_{Aav}}{B_{Bav}}=-\frac{1}{n}\sum_{k=1}^{n}\frac{\frac{d}{r}\cos\theta_{k}+1}{{\left(\frac{d}{r}\right)}^{2}+2\frac{d}{r}\cos\theta_{k}}$$

**Figure 5:**
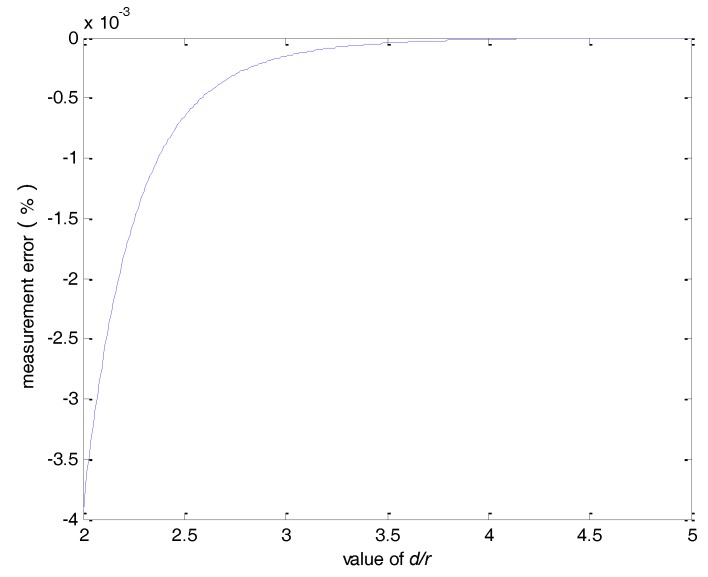
The measurement error verses the *d/r* ratio.

For *n* = 8, the measurement error as a function of the *d/r* ratio is shown in Figure 5. It can be seen that the error is less than 0.05% when *d*/*r* > 2.5.

From the above analysis, it can be seen that the measurement error is less than 0.05% when the number of sensors *n* is larger than 8, the distance between the conductors is 2.5 times the radius of sensor array, and *I*_A_ and *I*_B_ are equal in magnitude.

**Figure 6:**
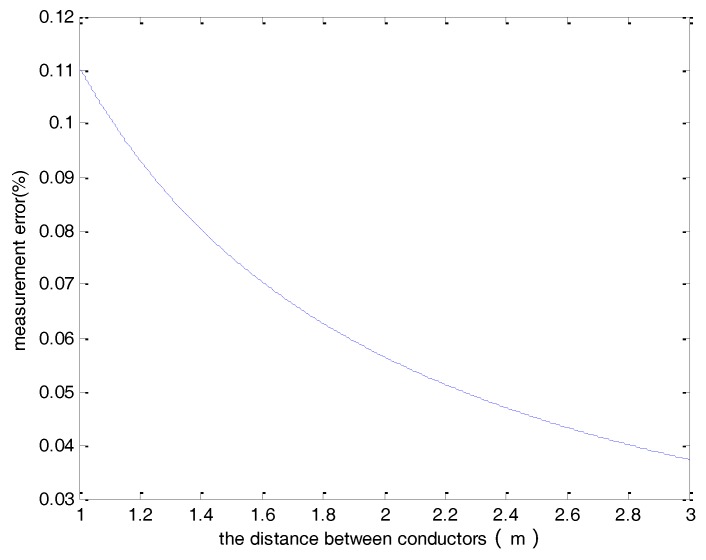
The measurement error of a single hall sensor verses the distance between conductors.

**Figure 7:**
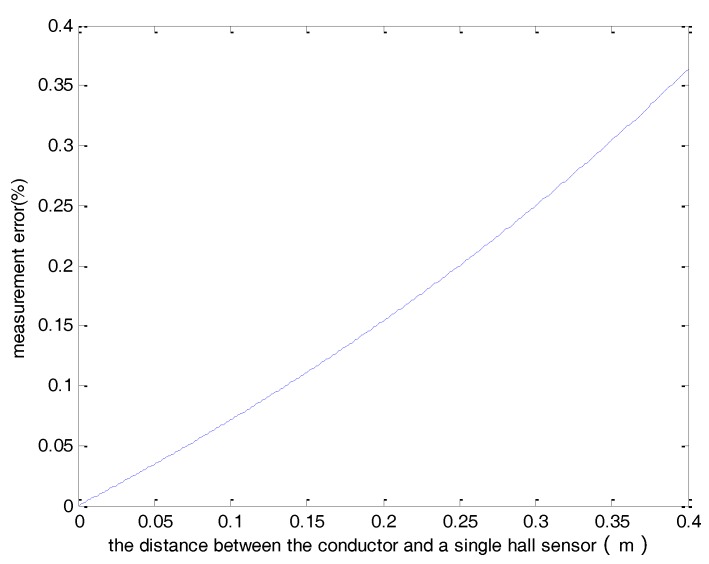
The measurement error of a single hall sensor verses the distance between the conductor and the sensor.

**Figure 8:**
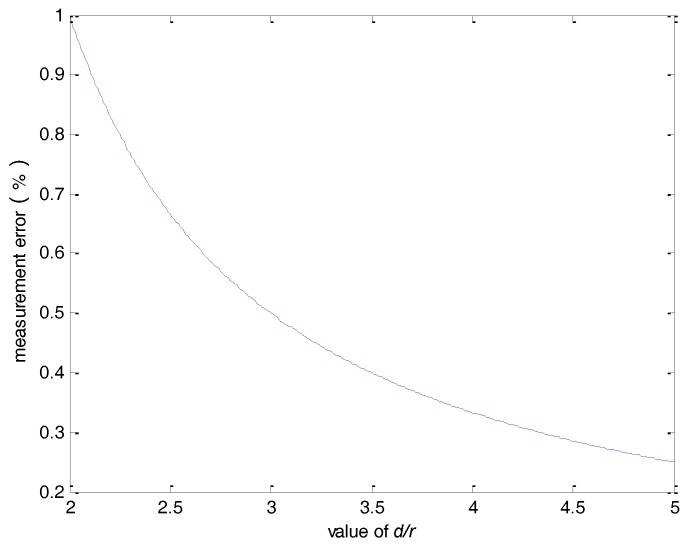
The measurement error of a single hall sensor verses the *d*/*r* ratio.

The average value of all hall sensors is used as the final measurement level. In this way, the effect of the external magnetic field interference can be reduced. Figure 6, Figure 7 and Figure 8 present the output results of a single hall sensor for the three cases investigated above. Comparing these results with the measurement error based on the average of all hall sensors (Figure 3, Figure 4 and Figure 5) reveals that by using the average value of the sensor array, the error can be effectively reduced.

## 3. Error Analysis of Off-Center Distance

The off-center position of the primary conductor is the most common problem that may lead to a significant measurement error when a current transformer is utilized to measure the current. A similar issue takes place when hall sensors are employed in this section; therefore, a compensation method of the magnetic field strength is proposed to improve the measurement accuracy of the hall sensors. The detailed analysis is presented below.

**Figure 9:**
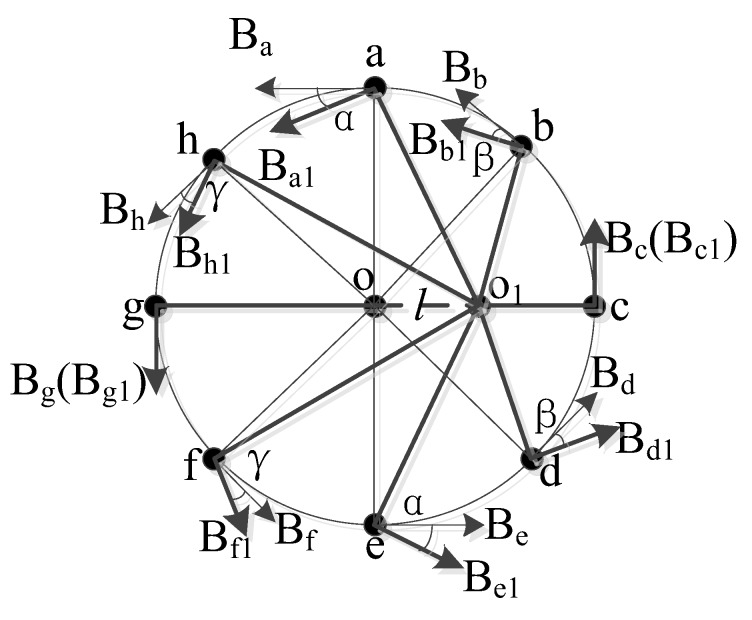
Schematic of an off-center primary conductor.

As shown in Figure 9, eight hall sensors are distributed evenly on a circle of radius *r* and center O at locations a, b, c, d, e, f, g, and h. When the primary conductor is shifted from O to O_1_, the off-center distance is *l*.

When the primary conductor is off-center, the magnetic field measured by the sensors at each point are *B_a_*, *B_b_*, *B_c_*, *B_d_*, *B_e_*, *B_f_*, *B_g_*, and *B_h_*, respectively, and the measured current can be calculated as:

(10) $$I_{1}=\frac{\left(B_{a}+B_{b}+B_{c}+B_{d}+B_{e}+B_{f}+B_{g}+B_{h}\right)}{8}\times 2\pi r$$

The measurement error is:

(11) $$I_{e1}=\frac{I-I_{1}}{I}\times 100\%$$

From (10) and (11), the measurement error without any improvement approach can be given as:

(12) $$I_{e1}=1-(\frac{2r^{2}-\sqrt{2}rl}{2\pi r(r^{2}+l^{2}-\sqrt{2}rl)}+\frac{2r^{2}+\sqrt{2}rl}{2\pi r(r^{2}+l^{2}+\sqrt{2}rl)}+\frac{r}{\pi (r^{2}+l^{2})}+\frac{1}{2\pi (r-l)}+\frac{1}{2\pi (r+l)})\times \frac{\pi r}{4}$$

**Figure 10:**
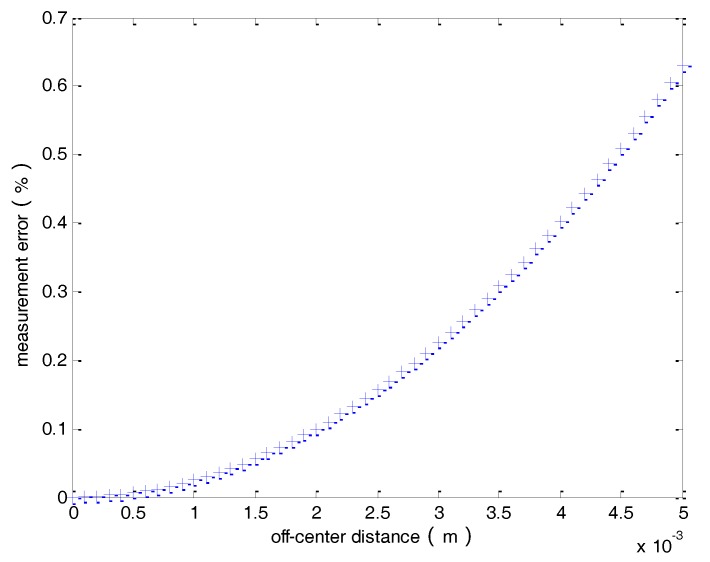
The measurement error verses the off-center distance.

Suppose the radius of the circle is *r* = 0.1 m and the maximum value of the off-center distance *l* is 5 mm. Then, the measurement error as a function of the off-center distance can be plotted as shown in Figure 10. It can be seen that the error increases as the off-center distance increases. The error is larger than 0.6% when the off-center distance is 5 mm.

It is obvious from Figure 10 and Equation (11) that the measured current *I*_1_ is less than the real current *I* due to the error introduced by the off-center distance. From Figure 10, one can conclude that if the magnetic field strength measured by magnetic field sensors is stronger, the measured current *I*_1_ will be larger and closer to the real current *I*. Hence, a compensation method for the magnetic field strength is proposed in this study to improve the measurement accuracy when the primary conductor is off-center. The detailed analysis is as follows.

As shown in Figure 9, when the conductor is off-center, taking point a as an example, the magnetic field strength measured at point a is *B_a_*. To make *B_a_* larger and measurement error smaller, we compensated for the magnetic field strength at point a, turning *B_a_* into *B_a_*_1_. 

**Figure 11:**
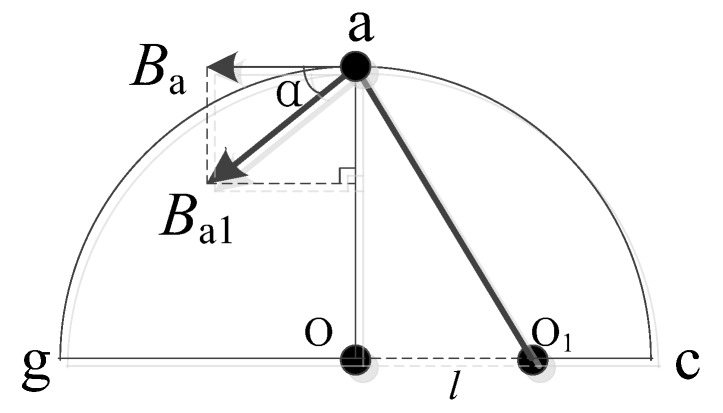
Schematic diagram of *B_a_* and *B_a_*_1_

As shown in Figure 11, the relationship between *B_a_* and *B_a_*_1_ is:

(13) $$B_{a1}=\frac{B_{a}}{\cos\alpha }$$

For the eight points sensors locations in Figure 9, the compensated magnetic fields can be written as:

(14) $$\left\{ \begin{array}{l} B_{a1}=\frac{B_{a}}{\cos\alpha }\\ B_{b1}=\frac{B_{b}}{\cos\beta }\\ B_{c1}=B_{c}\\ B_{d1}=\frac{B_{d}}{\cos\beta }\\ B_{e1}=\frac{B_{e}}{\cos\alpha }\\ B_{f1}=\frac{B_{f}}{\cos\gamma }\\ B_{g1}=B_{g}\\ B_{h1}=\frac{B_{h}}{\cos\gamma } \end{array}\right.$$

In (13) and (14), the distance *l* can be obtained by the magnetic field strength at points c and g, after which the angles of α, β, and γ can be calculated by using the radius *r*. 

By using this compensation method, the improved equation for the measured current can be given as:

(15) $$I_{2}=\frac{\left(B_{a1}+B_{b1}+B_{c1}+B_{d1}+B_{e1}+B_{f1}+B_{g1}+B_{h1}\right)}{8}\times 2\pi r$$

The measurement error is:

(16) $$I_{e2}=\frac{I-I_{2}}{I}\times 100\%$$

From (15) and (16), the measurement error using the proposed improvement approach can be given as:

(17) $$I_{e2}=1-\frac{\pi r}{4}\times (\frac{1}{\pi \sqrt{l^{2}+r^{2}}}+\frac{1}{\pi \sqrt{{\left(\frac{\sqrt{2}}{2}r-l\right)}^{2}+{\left(\frac{\sqrt{2}}{2}r\right)}^{2}}}+\frac{1}{\pi \sqrt{{\left(\frac{\sqrt{2}}{2}r+l\right)}^{2}+{\left(\frac{\sqrt{2}}{2}r\right)}^{2}}}+\frac{1}{2\pi (r-l)}+\frac{1}{2\pi (r+l)})$$

**Figure 12:**
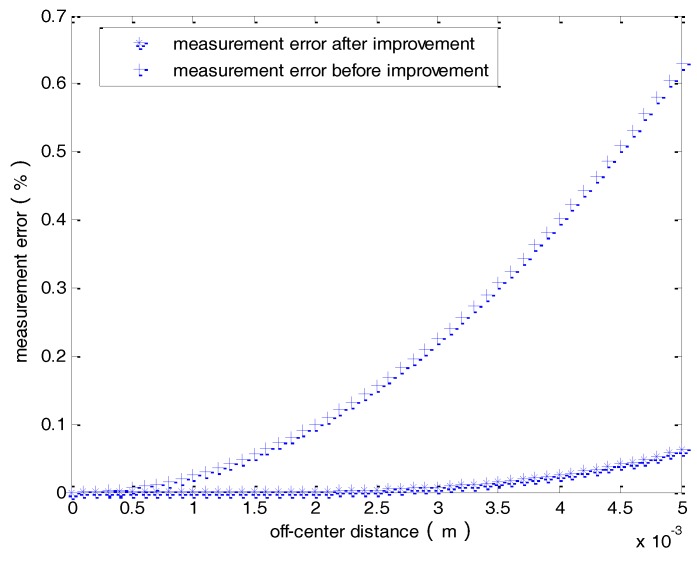
Comparison of the measurement errors without and with the compensation approach.

Figure 12 shows a comparison of the measurement error as a function of the off-center distance with and without the proposed compensation approach. By adopting the proposed compensation approach, the measurement error can be effectively reduced to a level less than 0.1%, even with a maximum off-center distance.

## 4. Performance Test

To assess the robustness of the proposed approach, a circular magnetic field sensor array with a radius of 0.1 m and eight hall sensors distributed evenly on a circle was developed and subjected to the below analysis. The type of the hall sensor employed was EQ-730L, produced by AKM, Tokyo, Japan, which has wide range of measurement and high sensitivity.

### 4.1. Influence of Magnetic Field due to a Parallel Conductor

**Figure 13:**
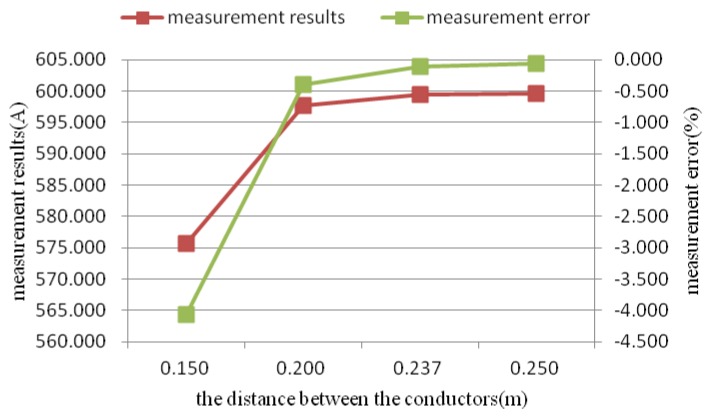
Measured results verses the distance between the conductors.

The developed sensor array was utilized to measure a current of 600 A. The overall measured value and the measurement error for various distances between the two conductors are shown in Figure 13. It can be seen that when the distance was larger than 0.237 m, i.e., when *d*/*r* was larger than 2.37, the measurement error was less than 0.1%. In the actual situation, the distance between the two conductors is much larger than 0.237 m, so the accuracy can meet the requirements of the 0.1 accuracy class.

### 4.2. Off-Center Conductor Analysis

**Figure 14:**
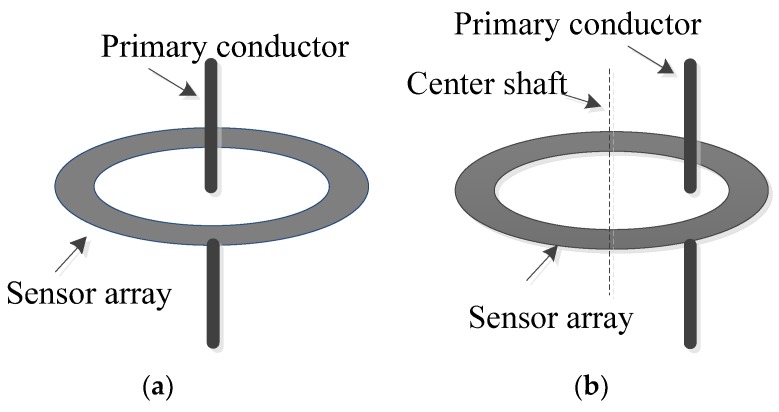
A schematic diagram for the primary conductor alignment with respect to the sensor array: (**a**) primary conductor is on the array’s center; (**b**) primary conductor is off-center.

Figure 14 shows the possible alignment of the primary conductor that could be perfectly aligned at the center of the sensor array or off-center. Measurements were conducted when the primary conductor was off-center, and the results are listed in Table 1. Without the proposed compensation approach, it can be seen that the measurement error was larger than 0.6%; however, it was reduced to 0.1% by the proposed approach.

### 4.3. Basic Accuracy Test

**Figure 15:**
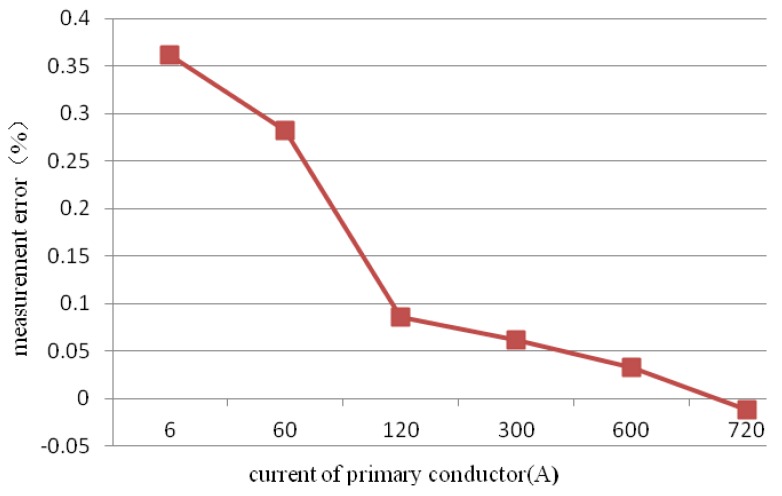
Basic accuracy test.

A basic accuracy test of the proposed current measurement method was carried out to measure the wide range of current levels. Figure 15 shows that the measurement error was less than 0.1% when the range of current was 20% to 120% of the investigated rated current (600 A).

Uncertainty in the measurements can be calculated as:

(18) $$\left\{ \begin{array}{l} s=\sqrt{\frac{\sum_{k=1}^{N}{\left(I_{\varepsilon k}-\bar{I_{\varepsilon }}\right)}^{2}}{N-1}}\\ u=\frac{s}{\sqrt{N}} \end{array}\right.$$

where *s* is the experimental standard deviations, *N* is the number of tests, $I_{\varepsilon k}$ is the measurement error of the *k*th test, $\bar{I_{\varepsilon }}$ is the average of the all measurement errors, and *u* is the measurement uncertainty.

Measurements of the 600 A current were repeated 10 times, and the uncertainty in the measurements was found to be only 0.033%.

## 5. Conclusions

In order to overcome the deficiency of conventional transformers and improve the accuracy of DC measurements, this paper proposes a current measurement method based on a circular magnetic field sensing array. By employing eight hall sensors distributed evenly on a circle and using the average value of these hall sensors as the final measured value, the interference generated by the external magnetic field can be effectively reduced and the measurement accuracy can be improved. Also, a kind of hall sensor compensation method is adopted to reduce the measurement error to less than 0.1%.

## Figures and Tables

**Table 1:** Measurement results for various off-center distances before and after improvement.

Current of Primary Conductor (A)	Off-Center Distance (mm)	Measurement Results before Improvement (A)	Measurement Results after Improvement (A)	Measurement Error before Improvement (%)	Measurement Error after Improvement (%)
600	1	599.85	599.99	0.025	0.001
600	2	599.34	599.93	0.110	0.012
600	3	598.74	599.89	0.210	0.019
600	4	597.42	599.78	0.430	0.036
600	5	596.16	599.63	0.640	0.062
